# Sexual orientation, crime victimization, and relationship to the offender: Insights from New Zealand police records, 2014–2024

**DOI:** 10.1016/j.puhe.2025.106027

**Published:** 2025-11-22

**Authors:** A.L. Gilmour, B.A. Feinstein, A. Plum

**Affiliations:** a Department of Psychology, Rosalind Franklin University of Medicine and Science, North Chicago, IL, USA; b NZ Policy Research Institute, Auckland University of Technology, New Zealand

**Keywords:** LGBT, Health disparities, Crime victimization

## Abstract

**Objectives:**

To examine sexual orientation differences in crime victimization rates by strangers and known offenders in a nationally representative sample of 2.58 million cisgender New Zealand (N.Z.) residents.

**Study design:**

We linked N.Z. Census data and police records from 2014 to 2024 to examine sexual orientation differences in rates of crime victimization (any crime, sexual assault, crime with a weapon, violence, and serious violence) separately for cisgender men and women. We further examined rates of each crime offence by strangers versus known offenders by sexual orientation, stratified by gender.

**Methods:**

We used logistic regression stratified by gender, adjusting for demographic covariates.

**Results:**

Sexual minority (SM) individuals faced higher victimization risk across categories of crime compared to heterosexual people, with the most consistent heightened risk observed for sexual assault. Bisexual people experienced heightened risk of victimization from both strangers and known offenders, whereas homosexual men generally experienced heightened risk of victimization from known offenders and homosexual women from strangers.

**Conclusions:**

There are differences in risk of being offended by strangers and known individuals by sexual orientation, with particularly stark disparities observed for women and bisexual individuals. Policymakers and social service organizations should consider the unique vulnerabilities of subsets of SM individuals when implementing crime prevention strategies.

## 1. Introduction

Sexual minority (SM; e.g., lesbian, gay, bisexual) individuals experience heightened rates of crime victimization compared to heterosexual individuals.^1–6^ Crime victims commonly report health difficulties,^7–10^ and crime is estimated to cost the United States (U.S.) between $450 billion and $3.2 trillion annually.^11^ To improve policies seeking to prevent crime and its consequences, it is important to determine subsets of the population at increased risk for specific crimes, and to explore other characteristics of crimes, such as victims’ relationship with the offender.

The majority of studies that have documented heightened crime victimization among SM individuals have used community-based samples, limiting their generalizability.^4^ However, one U.S. population-based study found that sexual and gender minority (SGM) people were five times more likely to be victims of violent crime than cisgender, heterosexual individuals.^5^ Few studies have broken down differences in crime victimization by subsets of the SM population (e.g., lesbian/gay vs. bisexual), making it challenging to determine who is at highest risk. In an exception, data from the British Crime Survey revealed that both lesbian/gay and bisexual individuals were at heightened risk for a variety of crimes, but odds were particularly high for bisexual individuals.^6^ In addition, one study using data from the U.S. National Crime Victimization Survey (NCVS) found that gay and bisexual males had higher odds of experiencing serious violence (ORs = 2.8 and 6.1, respectively) and any kind of violent crime (ORs = 2.0 and 4.0, respectively) within the past 6 months than heterosexual males.^1^ Bisexual males also had higher odds of experiencing aggravated (OR = 5.1) and simple assault (OR = 2.8), and gay males had higher odds of experiencing sexual assault (OR = 19.2). Lesbian females had higher odds of experiencing any kind of violent crime (OR = 3.7) and simple assault (OR = 5.4) than heterosexual females, whereas bisexual females experienced heightened rates of all crimes investigated (ORs = 6.9 to 12.6).^1^ The authors noted that some comparisons that yielded high odds ratios did not reach statistical significance due to the low sample sizes of specific SM groups,^1^ highlighting the need for continued research into disparities in rates of violence using large, population-based samples.

It is also important to understand *who* is perpetrating crimes against SM people. One study using data from the NCVS found that SGM, but not heterosexual, individuals were more likely to experience crime victimization by a known individual than a stranger.^12^ Another study using the NCVS found sexual orientation differences in rates of violence perpetrated by intimate partners (i.e., IPV), other known persons (e.g., friends, acquaintances, family members), and strangers.^1^ Gay and bisexual males were more likely than heterosexual males to experience violence from other known persons (ORs = 3.0 and 7.0, respectively). Lesbian females were more likely than heterosexual females to experience violence from other known persons (OR = 3.0) and strangers (OR = 8.8), and bisexual females were more likely to experience violence from intimate partners, other known persons, and strangers (ORs = 7.0 to 15.8).^1^ Again, some of the comparisons were underpowered, and this study examined the victims’ relationship to the offender collapsed across all types of crime.^1^ Nevertheless, these results speak to the nuances in who perpetrates crimes against SM individuals, suggesting that different crime prevention strategies may be warranted for different SM subgroups.

Population-based data collected in New Zealand (N.Z.) presents an excellent opportunity to explore sexual orientation disparities in crime victimization due to several unique data collection methods. Statistics New Zealand (StatsNZ) maintains a repository of data that includes the population-wide Census and data collected by government agencies in their Integrated Data Infrastructure (IDI). In 2023, the N.Z. Census began collecting data on sexual orientation and gender identity among individuals aged 16 and older. By linking these data to police victimization records, we are able to examine rates of crime victimization across subsets of the SM population relative to heterosexual individuals among nearly 2.58 million N.Z. residents. These records also include the relationship between the victim and offender, enabling detailed comparisons by sexual orientation.

N.Z. is considered an inclusive country for SGM people, and was named the tenth most socially accepting country of SGM individuals out of 175 countries in 2021.^13^ Despite this, N.Z. population-based research found that 45.5 % of SGM individuals reported experiencing crime victimization in the past 12 months, compared to 31.2 % of cisgender, heterosexual individuals.^14^ However, prior crime victimization research has relied on self-report methods (e.g., interviews, questionnaires), which are prone to recall bias.^15^ By contrast, police victimization records include crimes that people may not self-report and detailed information reported at the time of the crime. Although there is evidence that individuals frequently do not report crime victimization to the police,^16^ prior research in N.Z. did not find significant differences in likelihood of reporting crime to the police between SGM and cisgender, heterosexual individuals,^14^ making the present data a valuable source of information in comparing the odds of victimization by sexual orientation.

Therefore, the aims of the present study were to examine sexual orientation differences in: 1) rates of different categories of crimes (i.e., any crime, sexual assault, violence, serious violence, and crime with a weapon) as recorded by the N.Z. police separately among cisgender men and women across ten years (2014–2024), and 2) rates of each of these crimes perpetrated by known offenders and strangers. For crimes categorized as violence or serious violence, we further broke the category of known offenders down into parents, friends, or romantic partners (current/former).

## 2. Methods

### 2.1. Participants

The data for the present study were drawn from the IDI, as described above. Participants were categorized as ‘heterosexual’, ‘homosexual’, ‘bisexual’, or ‘sexual identity not elsewhere classified’ (i.e., ‘other’), which were the response items included in the 2023 population-wide Census. The Census provides individual-level characteristics (e.g., sex, gender identity, age, partnership status, ethnicity, highest post-school qualification) and residential characteristics (e.g., region, urban/rural status, and deprivation score, which runs from 1 for the least deprived areas to 10 for the most deprived areas for each meshblock, the smallest geographic unit). We restricted our sample to cisgender individuals (i.e., individuals whose gender identity matched their sex assigned at birth) ages 16 and older at the time of the 2023 Census due to small sample sizes for specific types of crime victimization at the intersections of sexual orientation and transgender/nonbinary identities.

We then linked our sample to N.Z. police victimization records between July 2014 and 2024. Records include the offence type, reporting date, relationship between offender and victim, and whether a weapon was involved. Each offence was categorized according to the Australian and New Zealand Standard Offence Classification (ANZSOC) as well as police offence codes, which describe the offence type in detail. A seriousness score was provided for each offence, calculated by the average number of days of imprisonment imposed on every offender convicted of that offence. We examined the following crime types: any crime, sexual assault, crimes involving a weapon, violent offence, and serious violent offence with a seriousness score ≥250. We also examined rates of each crime committed by a stranger or known person (i.e., family members, caregivers, current/former partners, friends, and flatmates). For violent and serious violent offences, we further broke down known persons into parent, current/former partner, or friend. Due to lower prevalence rates, we were not able to examine specific known offenders for sexual assault and crimes involving a weapon.

To ensure that all individuals in our sample were present in N.Z. for a comparable length of time, we linked our sample to border movement data, which provides date stamps each time an individual exits or enters N.Z. We restricted our sample to individuals who spent <700 days overseas in the 10-year period. Our final dataset included almost 2.58 million individuals. For more information about the IDI and dataset, please see the Appendix.

### 2.2. Data analysis

We used logistic regression to (a) estimate differences in the likelihood of reporting crime victimization to the police by sexual orientation, regressing crime victimization on sexual orientation separately for each type of crime and (b) examine differences in the likelihood of crime being perpetrated by a known offender and a stranger by sexual orientation. Within each crime type, we regressed crime perpetration by a known offender and crime perpetration by a stranger on sexual orientation. For violent and serious violent offences, we also examined likelihood of crime perpetration by specific known people: parents, current/former partners, and friends. Sexual orientation was dummy coded with heterosexual as the reference category. Analyses were run separately for cisgender men and women, controlling for age, deprivation score, ethnicity, region, highest post-school qualification, partnership status, and urban-rural marker.

## 3. Results

Table 1 provides descriptive statistics by sexual orientation, stratified by gender. Table 2 provides results from logistic regressions examining likelihood of experiencing any crime, sexual assault, crime with a weapon, and likelihood of experiencing each crime by a stranger and known person. Compared to heterosexual women, likelihood of crime victimization was higher among homosexual women (OR = 1.25), bisexual women (OR = 1.31), and women with other non-heterosexual identities (OR = 1.07), and bisexual men were more likely to experience crime victimization than heterosexual men (OR = 1.06). Specifically, homosexual women were more likely to be victimized by strangers (OR = 1.56) and less likely to be victimized by known persons (OR = 0.91). Bisexual women and men, by contrast, experienced higher risk of being victimized by strangers (ORs = 1.23 and 1.12, respectively) and known persons (ORs = 1.29 and 1.26, respectively) for any crime. Higher risks for sexual assault victimization were found for all non-heterosexual women and men, including when the offender was a stranger or known (ORs ranged from 1.43 to 6.82). Bisexual women were also more likely to be victims of a crime where a weapon was involved (OR = 1.30).

Table 3 provides results from logistic regressions examining likelihood of experiencing violence and serious violence, and likelihood of experiencing these crimes by a stranger, known person, and specific known persons. All SM groups experienced heightened risk of violence and serious violence except men with other non-heterosexual identities (ORs ranged from 1.06 to 1.47). Homosexual women were more likely to be violently and seriously violently offended by a stranger (ORs = 2.07 and 1.62, respectively) and less likely to be violently offended by a known person (OR = 0.91), whereas bisexual women experienced elevated risk of violence and serious violence from both strangers and known persons (ORs ranged from 1.13 to 1.81). Homosexual men were more likely to experience violence and serious violence by a known person (ORs = 1.26 and 1.72, respectively), whereas bisexual men experienced elevated risk of violence from strangers and known persons (ORs = 1.15 and 1.22, respectively), and serious violence from strangers (OR = 1.29).

## 4. Discussion

Our results add important nuance to previous population-based research. All SM women were at heightened risk for any crime victimization, violent crime, and serious violent crime compared to heterosexual women. Only bisexual men were at heightened risk for any crime victimization compared to heterosexual men, but both homosexual and bisexual men were at heightened risk for violent and serious violent crime. These results expand upon U.S. population-based research, which was unable to estimate the statistical significance of some differences in odds between heterosexual and specific SM groups due to small sample sizes.^1^

Consistent with prior research, sexual assault rates were elevated among all SM groups.^17^ Expanding on prior research, we found that all SM groups were significantly more likely than their heterosexual counterparts to experience sexual assault by strangers *and* known offenders. Although the reasons for these disparities are not clear, one pervasive risk factor for sexual assault in adulthood is childhood sexual abuse, which is elevated among SM individuals.^4^ Researchers have also suggested that internalized heterosexism, or the internalization of negative social attitudes toward SM individuals,^18^ may hinder SM individuals’ development of positive intimate relationships with others and impede sexual communication about preferences and desires, increasing risk for sexual assault.^19^

Our results aligned with prior research demonstrating that odds of crime victimization are particularly high among bisexual individuals.^6^ Bisexual individuals generally experienced heightened risk of crime perpetration from both strangers and known offenders. Although attitudes toward lesbian/gay individuals have improved, attitudes toward bisexuality remain negative to neutral at best.^20^ Bisexual individuals also experience discrimination from *both* heterosexual and lesbian/gay individuals,^20,21^ which may explain their heightened risk for crime victimization. We also found that bisexual individuals were at heightened risk of experiencing violence or serious violence by strangers, parents, friends, and current/former partners. Although we do not know if these were bias-motivated crimes, these results suggest that anti-bisexual violence may be pervasive across a variety of relationships.

The odds of experiencing various types of crime by a stranger and known offender were more nuanced among homosexual women and men. Homosexual men experienced less crime from strangers but more crime from known offenders, whereas the opposite pattern was observed for homosexual women. Homosexual men faced higher risk of violent and serious violent crime from all known offender types except friends committing serious violence. Homosexual women had higher risk of violent victimization from parents but lower risk for violent and serious violent victimization from current/former partners, which may explain their overall lower known-offender victimization rates. Men are more likely to perpetrate IPV than women,^17^ which may account for homosexual women’s lower risk of partner victimization.

### 4.1. Limitations and strengths

The results of the present study should be interpreted in light of several limitations. First, individuals reported their sexual orientation beginning in the 2023 Census; we used the sexual orientation they reported in 2023 to categorize their sexual orientation for the entire ten-year study period. Despite this limitation, the present results are striking in suggesting disparities by individuals’ current sexual orientation in their history of victimization over ten years. Second, we categorized individuals as having experienced each crime based on police victimization records. However, individuals do not always report crime to the police, suggesting that the victimization rates are likely underestimates. This may be especially true when the perpetrator is a same-gender partner or when there is stigma associated with a type of crime (e.g., sexual assault among men).^22^ For instance, prior research in N.Z. found that only 8.8 % of SGM people who had experienced a crime reported it to the police.^14^ The odds ratios in the present study may therefore underestimate the differences in rates of victimization between heterosexual and SM individuals.

Despite limitations, using police victimization records also represents a strength of our study. Prior research examining victimization rates by sexual orientation has relied on retrospective self-report interviews and questionnaires, which are prone to recall bias^15^ because many of these measures ask individuals to report crime across the past 6 or 12 months. By contrast, crimes are likely to be reported to the police soon after they occur. Additionally, police records include detailed information, which enabled us to assess type of crime and relationship to the offender with more specificity than previous research. Finally, linking these records to Census data provided us with a large, nationally representative dataset, which gave us greater statistical power to examine sexual orientation differences than previous studies. This is a strength of N.Z.’s unique data collection methods, which integrate Census data and data collected by government agencies; similar methods could be used in other countries to improve the rigor of their data on crime victimization.

### 4.2. Public health implications and future directions

Hate-motivated crimes against SM individuals are likely to account for some of their disparities in crime victimization, which has important health implications. Research has found that SGM crime victims who believed the crime was motivated by their identity experienced worse health outcomes than those who did not believe the crime was identity-motivated.^8^ Importantly, SM youth in schools with bullying policies that mention sexual orientation and gender identity are less likely to experience victimization,^23^ and those living in U.S. states with hate crime laws that name SM individuals as a protected group report fewer suicide attempts than those in other states.^24^ N.Z. laws name sexual orientation as an aggravating factor for crimes, but N.Z. does not have standalone hate crime laws, meaning that hate-motivated crimes are not treated as a separate category of offence.^25^ This poses barriers to tracking and understanding the effects of hate-motivated crimes in N.Z.,^26^ and may contribute to the prevalence of crime against SM individuals. Implementing hate crime laws and anti-bullying policies that explicitly prohibit victimization based on sexual orientation is a public health priority that may reduce violence against these groups.

Given that we found the starkest disparities in experiencing sexual violence, it is highly important to implement tailored sexual assault prevention programs for SM populations. Although few sexual assault prevention programs have been tailored to SM individuals, one pilot trial of a brief online program targeting risk factors for sexual violence (e.g., alcohol use, sexual risk behaviours) provided personalized feedback based on whether participants identified as SGM. The program resulted in lower sexual assault victimization at 3-month follow-up with no differences in effectiveness by SGM status, suggesting promise for a tailored and scalable approach to sexual assault prevention.^27^ However, the program did not provide further personalized feedback to participants based on their specific sexual orientation or gender identity. Our results revealed nuances in sexual assault and other crime victimization and relationship to the perpetrator by sexual orientation, suggesting that particular subgroups of the SM community may benefit from targeted prevention strategies.

Further, the present study suggests that many crimes against SM individuals are perpetrated by known individuals, including family members, parents, friends, and intimate partners. Although IPV is common among SM individuals,^28^ they face unique barriers to seeking help, including perceived lack of knowledge about SM issues in IPV organizations.^29^ As such, services that are tailored and advertise to SM individuals may reduce barriers to help-seeking. Finally, little is known about factors that put SM individuals at risk for crime perpetration by other types of known individuals. Research is needed to better understand these factors and their implications for policies and interventions.

## Supplementary Material

1

## Figures and Tables

**Table 1:** Socio-demographic characteristics of the 2023 Census sample and crime victimization by offence type as recorded by the N.Z. police from 2014 to 2024.

	Men					Women				
	Heterosexual	Homosexual	Bisexual	Other	Total	Heterosexual	Homosexual	Bisexual	Other	Total
Age	48.34 (19.44)	41.78 (16.94)	35.15 (17.05)	35.22 (16.9)	48.06 (19.36)	49.9 (19.22)	41.31 (17.35)	30.04 (12.33)	31.87 (14.27)	48.98 (18.93)
Deprivation score	5.21 (2.82)	5.45 (2.74)	5.73 (2.73)	6.04 (2.76)	5.22 (2.82)	5.24 (2.83)	5.54 (2.75)	5.78 (2.73)	5.86 (2.74)	5.27 (2.82)
Ethnicity										
European	0.690	0.668	0.706	0.641	0.690	0.689	0.697	0.650	0.653	0.687
Māori	0.137	0.167	0.162	0.229	0.138	0.140	0.198	0.234	0.219	0.144
Pacific People	0.048	0.043	0.036	0.050	0.048	0.049	0.032	0.038	0.037	0.048
Asian	0.114	0.106	0.086	0.064	0.113	0.113	0.064	0.067	0.078	0.110
MELAA/Other	0.011	0.016	0.010	0.016	0.011	0.009	0.009	0.011	0.013	0.011
Highest post-school qualification										
None	0.171	0.079	0.090	0.129	0.168	0.155	0.083	0.060	0.068	0.150
Below Bachelor	0.582	0.516	0.630	0.614	0.582	0.523	0.513	0.609	0.562	0.526
Bachelor+	0.171	0.079	0.090	0.129	0.168	0.155	0.083	0.060	0.068	0.150
Partnered	0.642	0.386	0.320	0.280	0.634	0.594	0.498	0.384	0.298	0.584
Major/large urban	0.616	0.772	0.755	0.736	0.621	0.624	0.685	0.746	0.763	0.630
Crime victimization by offence type as recorded by the New Zealand Police (2014–2024)										
Any crime	0.201	0.231	0.246	0.231	0.202	0.160	0.232	0.275	0.240	0.165
Sexual assault	0.002	0.009	0.007	0.007	0.002	0.012	0.026	0.054	0.046	0.013
Weapon involved	0.011	0.012	0.015	0.013	0.011	0.006	0.008	0.013	0.010	0.006
Violence	0.060	0.083	0.092	0.089	0.060	0.058	0.094	0.140	0.109	0.062
Serious violence	0.014	0.025	0.027	0.023	0.014	0.018	0.033	0.068	0.056	0.020
*N*	1,208,412	19,398	14,181	2946	1,244,937	1,267,572	15,867	47,340	8505	1,339,284

Abbreviation: MELAA, Middle Eastern Latin American, and African.

**Table 2:** Odd ratios on crime victimization (2014–2024) by sexual orientation and relationship to the offender.

Crime type	Men				Women			
	Heterosexual	Homosexual	Bisexual	Other	Heterosexual	Homosexual	Bisexual	Other
Any	*reference*	0.978 [0.945 1.012]	1.059 *** [1.018 1.101]	0.947 [0.868 1.034]	*reference*	1.249*** [1.202 1.298]	1.305*** [1.277 1.334]	1.066** [1.013 1.122]
by stranger	*reference*	0.915** [0.839 0.998]	1.121** [1.023 1.229]	0.854 [0.682 1.068]	*reference*	1.557*** [1.415 1.714]	1.227*** [1.155 1.303]	1.049 [0.906 1.214]
by known person	*reference*	1.295*** [1.173 1.43]	1.257*** [1.128 1.402]	0.961 [0.749 1.234]	*reference*	0.908** [0.829 0.994]	1.285*** [1.231 1.341]	0.964 [0.865 1.074]
Sexual assault	*reference*	4.850*** [4.146 5.674]	2.637*** [2.159 3.221]	2.364*** [1.532 3.646]	*reference*	1.435*** [1.297 1.589]	1.982*** [1.895 2.073]	1.761*** [1.586 1.954]
by stranger	*reference*	6.818*** [3.473 13.386]	4.029*** [1.83 8.87]	—^a^	*reference*	1.719*** [1.225 2.412]	1.770*** [1.495 2.096]	1.731*** [1.186 2.528]
by known person	*reference*	4.271*** [3.075 5.932]	1.970*** [1.267 3.062]	3.008*** [1.422 6.364]	*reference*	1.433*** [1.174 1.75]	2.019*** [1.854 2.198]	1.536*** [1.237 1.908]
Weapon involved	*reference*	0.963 [0.845 1.097]	1.118 [0.974 1.283]	0.857 [0.623 1.177]	*reference*	1.058 [0.889 1.259]	1.299*** [1.191 1.416]	0.978 [0.783 1.223]
by stranger	*reference*	0.802 [0.609 1.056]	1.236 [0.957 1.596]	0.659 [0.313 1.385]	*reference*	1.354 [0.924 1.986]	1.107 [0.878 1.396]	1.357 [0.838 2.197]
by known person	*reference*	1.310** [1.033 1.662]	0.904 [0.658 1.243]	1.078 [0.609 1.905]	*reference*	0.945 [0.709 1.26]	1.263*** [1.102 1.447]	0.848 [0.583 1.234]
*N*	1,208,412	19,398	14,181	2946	1,267,572	15,867	47,340	8505

Notes: Numbers in [] refer to 95 % confidence interval;

***, **, * indicate significance at the 1 %, 5 %, 10 % level. Known persons include parents and step-parents, siblings, children, caregivers, current/former partners, flatmates, and friends. Analyses controlled for demographic covariates (age, deprivation score, ethnicity, region, highest post-school qualification, partnership status, urban-rural marker).

a There were not sufficient numbers of cases of sexual assault by strangers to estimate the odds ratio for men of other sexual orientations

**Table 3:** Odd ratios of violent and serious violent crime victimization (2014–2024) by sexual orientation and relationship to the offender.

Crime type	Men				Women			
	Heterosexual	Homosexual	Bisexual	Other	Heterosexual	Homosexual	Bisexual	Other
Violence	*reference*	1.181*** [1.12 1.245]	1.158*** [1.092 1.227]	1.008 [0.886 1.147]	*reference*	1.216*** [1.15 1.287]	1.411*** [1.371 1.452]	1.064* [0.991 1.142]
by stranger	*reference*	0.963 [0.858 1.081]	1.146** [1.016 1.292]	0.824 [0.609 1.115]	*reference*	2.072*** [1.824 2.354]	1.323*** [1.217 1.439]	1.130 [0.921 1.385]
by known person	*reference*	1.255*** [1.128 1.396]	1.220*** [1.086 1.37]	0.996 [0.769 1.288]	*reference*	0.913* [0.831 1.003]	1.276*** [1.222 1.333]	0.961 [0.861 1.074]
by parent	*reference*	1.763*** [1.243 2.5]	1.403** [1.009 1.953]	0.681 [0.254 1.822]	*reference*	1.355** [1.014 1.809]	1.400*** [1.229 1.596]	1.649*** [1.241 2.192]
by current/former partner	*reference*	1.648*** [1.327 2.048]	1.276 [0.953 1.708]	0.705 [0.335 1.486]	*reference*	0.441*** [0.372 0.522]	1.131*** [1.065 1.201]	0.764*** [0.65 0.897]
by friend	*reference*	1.622** [1.116 2.358]	1.478* [0.973 2.246]	1.090 [0.407 2.918]	*reference*	1.297 [0.848 1.983]	1.557*** [1.273 1.904]	1.188 [0.711 1.986]
Serious violence	*reference*	1.471*** [1.340 1.615]	1.325*** [1.195 1.47]	1.066 [0.838 1.355]	*reference*	1.077** [1.008 1.151]	1.422*** [1.378 1.468]	1.097** [1.015 1.187]
by stranger	*reference*	1.121 [0.911 1.379]	1.292** [1.044 1.6]	0.552* [0.275 1.106]	*reference*	1.621*** [1.276 2.058]	1.678*** [1.481 1.901]	1.573*** [1.182 2.093]
by known person	*reference*	1.719*** [1.392 2.121]	1.128 [0.857 1.483]	1.399 [0.854 2.294]	*reference*	1.137 [0.966 1.339]	1.630*** [1.519 1.749]	1.235 [1.03 1.479]
by parent	*reference*	3.334** [1.337 8.315]	1.707 [0.535 5.443]	2.506 [0.347 18.074]	*reference*	1.426 [0.82 2.48]	1 779*** [1.404 2.255]	1.465 [0.803 2.673]
by current/former partner	*reference*	3.584*** [2.195 5.851]	2.304** [1.13 4.695]	—^a^	*reference*	0.444*** [0.304 0.65]	1.415*** [1.261 1.586]	0.989 [0.729 1.34]
				—^a^				
by friend	*reference*	1.114	1.545	—^a^	*reference*	0.856	1.812***	1.624
		[0.411 3.022]	[0.63 3.788]	—^a^		[0.318 2.303]	[1.282 2.56]	[0.719 3.67]
*N*	1,208,412	19,398	14,181	2946	1,267,572	15,867	47,340	8505

Notes: Numbers in [] refer to 95 % confidence interval;

***, **, * indicate significance at the 1 %, 5 %, 10 % level. Known persons include parents and step-parents, siblings, children, caregivers, current/former partners, flatmates, and friends. Serious violence is defined as an offence with a seriousness score of 250 or higher (for reference: common manual assault has a seriousness score of 17.83). Analyses controlled for demographic covariates (age, deprivation score, ethnicity, region, highest post-school qualification, partnership status, urban-rural marker).

a There were not sufficient numbers of cases of serious violence by current/former partners and friends to estimate odds ratios for men of other sexual orientations.

## Appendix A. Supplementary data

Supplementary data to this article can be found online at https://doi.org/10.1016/j.puhe.2025.106027.
