# Supplementary material for: Sexual orientation, crime victimization, and relationship to the offender: Insights from New Zealand police records, 2014–2024
Source: Public Health. Author manuscript; Available in PMC 2026 Jun 22. (PMC13286289; doi:10.1016/j.puhe.2025.106027)
Supplement: 1 [file NIHMS2186757-supplement-1.docx]

**Appendix**

*Information about the Integrated Data Infrastructure (IDI)*

Using a probabilistic model, StatsNZ uses individual-specific credentials (e.g., name, birth date) to identify the same individual across different datasets and then assigns a unique identifier to each individual. Identifiable characteristics are then removed (e.g., name) or made confidential (e.g., exact birth date changed to birth month) before researchers can work with the data. According to StatsNZ, the link rate between the datasets is above 90%, while the estimated false positive rate (a record is linked to the wrong person) is below 2%. To access the IDI, researchers must submit a detailed project application that demonstrates public value and community engagement. Only approved projects and researchers are granted access, and access can only occur in secure “Data Labs” across New Zealand with strict rules around data confidentiality, output checking, and prohibition of identifying information.

*Information about Categorizing Offences*

Each offence is categorised according to the Australian and New Zealand Standard Offence Classification (ANZSOC), and the following offence types can be found in the records: homicide; assault; sexual offences; robbery, blackmail, and extortion; burglary; theft; fraud and related offences. Additionally, the record includes police offence codes, which describe the offence type on a very granular level. There are, in total, around 700 unique police offence codes. These police offence codes in combination with the ANZSOC codes were used to classify the offence type in this study. Moreover, every police offence code has a seriousness score,^[[1]](#footnote-1)^ which is calculated by the average number of days of imprisonment imposed on every offender convicted of that offence.

Definition of violence is based on the inclusion of the following offence types (where possible, also providing the Australian and New Zealand Standard Offence Classification (ANZSOC) group code):

- Murder (ANZSOC: 0111)
- Attempted Murder (0121)
- Manslaughter (0131)
- Assault not further defined (0210)
- Common assault (0213)
- Aggravated sexual assault (0311)
- Non-aggravated sexual assault (0312)
- Abduction and kidnapping (0511)
- Aggravated robbery (0611)
- Non aggravated robbery (by threats to person)
- Blackmail and extortion (0621)
- Committing burglary with a weapon (firearm or other weapon, other aggravated burglary offences, remained after burglary, remained with intent)

Examples of offences with the corresponding seriousness score [police offence code]:

- Theft (under $500) [4373]: 25.15
- Common assault (manually) [1653]: 17.83
- Common assault (stabbing/cutting weapon) [1594]: 57.65
- Assaults with intent to injure (other weapon) [1522]: 151.57
- Aggravated injury (manually) [1436]: 531.68
- Injures intent to great bodily harm (stabbing/cutting weapon) [1427]: 923.89

*StatsNZ disclaimer for output produced from the Integrated Data Infrastructure (IDI)*

These results are not official statistics. They have been created for research purposes from the Integrated Data Infrastructure (IDI) which is carefully managed by StatsNZ. For more information about the IDI please visit https://www.stats.govt.nz/integrated-data/.

*StatsNZ disclaimer for Census 2023 data*

Access to the data used in this study was provided by Stats NZ under conditions designed to give effect to the security and confidentiality provisions of the Data and Statistics Act 2022. The results presented in this study are the work of the author, not StatsNZ or individual data suppliers.

1. For details, see <https://www.corrections.govt.nz/resources/statistics/corrections-volumes-report/2007/10-data-definitions-and-groupings/10.6-offence-category-charge-category>. [↑](#footnote-ref-1)
